# Supplementary material for: A Ternary Copper (II) Complex with 4-Fluorophenoxyacetic Acid Hydrazide in Combination with Antibiotics Exhibits Positive Synergistic Effect against Salmonella Typhimurium
Source: Antibiotics (Basel). 2022 Mar 15;11(3):388. doi: 10.3390/antibiotics11030388 (PMC8944508; doi:10.3390/antibiotics11030388)
Supplement: Supplementary file 1 [file antibiotics-11-00388-s001.zip › Sup File S1.pdf]

Experiment Results

Date: 26-01-2021
Operator:
Array: Check-MDR CT103XL.arr
Output from: Check-Points 4.9.0.61 (16-10-2017)

CT 103XL Check-MDR

Sample name	CARBA	ESBL	AMPC	CARBA-gene	AMPC-gene	CTX-M group	SHV SNP	TEM SNP	Other
CPTube 32036481									
sample 1	N	N	N	-	-	-	-	-	-
CPTube 32036482									
sample 2	N	N	N	-	-	-	-	-	-
CPTube 32036483									
sample 3	N	N	N	-	-	-	-	-	-
CPTube 32036484									
sample 4	N	N	N	-	-	-	-	-	-
CPTube 32036485									
sample 5	N	N	N	-	-	-	-	-	-
CPTube 32036486									
sample 6	N	N	N	-	-	-	-	WT	-
CPTube 32036487									
sample 7	N	N	N	-	-	-	-	WT	-
CPTube 32036488									
sample 8	N	N	N	-	-	-	-	WT	-
CPTube 32036489									
sample 9	N	N	N	-	-	-	-	-	-
CPTube 32036490									
sample 10	N	N	N	-	-	-	-	WT	-
CPTube 32036491									
sample 11	N	N	N	-	-	-	-	-	-

Experiment Results

Date: 26-01-2021
Operator:
Array: Check-MDR CT103XL.arr
Output from: Check-Points 4.9.0.61 (16-10-2017)

Sample name	CARBA	ESBL	AMPC	CARBA-gene	AMPC-gene	CTX-M group	SHV SNP	TEM SNP	Other
CPTube 32036492									
sample 12	N	N	N	-	-	-	-	-	-
CPTube 32036493									
sample 13	N	N	N	-	-	-	-	WT	-
CPTube 32036494									
sample 14	N	N	N	-	-	-	-	WT	-
CPTube 32036495									
sample 15	N	N	N	-	-	-	-	WT	-
CPTube 32036496									
sample 16	N	N	N	-	-	-	-	-	-
CPTube 32036497									
sample 17	N	Y	N	-	-	2	-	-	-
CPTube 32036498									
sample 18	N	N	N	-	-	-	-	WT	-
CPTube 32036499									
sample 19	N	N	N	-	-	-	-	WT	-
CPTube 32036500									
sample 20	N	N	N	-	-	-	-	-	-
CPTube 32036501									
sample 21	N	N	N	-	-	-	-	-	-
CPTube 32036502									
sample 22	N	N	N	-	-	-	-	-	-

Experiment Results

Date: 26-01-2021
Operator:
Array: Check-MDR CT103XL.arr
Output from: Check-Points 4.9.0.61 (16-10-2017)

CT 103XL Check-MDR

Sample name	CARBA	ESBL	AMPC	CARBA-gene	AMPC-gene	CTX-M group	SHV SNP	TEM SNP	Other
CPTube 32036503									
sample 23	N	N	N	-	-	-	-	WT	-
CPTube 32036504									
sample 24	N	N	N	-	-	-	-	WT	-
CPTube 32036390									
sample 25	N	N	N	-	-	-	-	-	-
CPTube 32036391									
sample 26	N	N	N	-	-	-	-	-	-
CPTube 32036392									
sample 27	N	N	N	-	-	-	-	WT	-
CPTube 32036393									
sample 28	N	N	N	-	-	-	-	WT	-
CPTube 32036396									
sample 29	N	N	N	-	-	-	-	WT	-
CPTube 32036414									
30 repetition	N	N	N	-	-	-	-	-	-
CPTube 32036398									
sample 31	N	N	N	-	-	-	-	-	-
CPTube 32036399									
sample 32	N	N	N	-	-	-	-	WT	-
CPTube 32036400									
sample 33	N	N	N	-	-	-	-	-	-

Experiment Results

Date: 26-01-2021
Operator:
Array: Check-MDR CT103XL.arr
Output from: Check-Points 4.9.0.61 (16-10-2017)

CT 103XL Check-MDR

Sample name	CARBA	ESBL	AMPC	CARBA-gene	AMPC-gene	CTX-M group	SHV SNP	TEM SNP	Other
CPTube 32036401									
sample 34	N	N	N	-	-	-	-	-	-
CPTube 32036402									
sample 35	N	N	N	-	-	-	-	-	-
CPTube 32036403									
sample 36	N	N	N	-	-	-	-	-	-
CPTube 32036404									
sample 38	N	N	N	-	-	-	-	-	-
CPTube 32036405									
sample 37	N	N	N	-	-	-	-	-	-
CPTube 32036406									
sample 39	N	N	N	-	-	-	-	WT	-
CPTube 32036407									
sample 40	N	N	N	-	-	-	-	-	-
CPTube 32036410									
sample 41	N	N	N	-	-	-	-	-	-
CPTube 32036411									
sample 42	N	N	N	-	-	-	-	-	-
CPTube 32036412									
sample 43	N	N	N	-	-	-	-	WT	-
