# Supplementary material for: A Ternary Copper (II) Complex with 4-Fluorophenoxyacetic Acid Hydrazide in Combination with Antibiotics Exhibits Positive Synergistic Effect against Salmonella Typhimurium
Source: Antibiotics (Basel). 2022 Mar 15;11(3):388. doi: 10.3390/antibiotics11030388 (PMC8944508; doi:10.3390/antibiotics11030388)
Supplement: Supplementary file 1 [file antibiotics-11-00388-s001.zip › Tables Sup-correction1.pdf]

Full names of antibiotics used in this study: AMP – ampicillin; MER – meropenem; COL – colistin; CFT – ceftriaxone; CIP – ciprofloxacin; SUL – sulfisoxazole; TET – tetracycline; DRI-12 – copper complex [Cu(4-FH)(phen)(ClO_4)^2]

Suplementary Tables:

Suplementar Table S1: Antimicrobial resistance profiles in ST isolated from food and humans in Brazil from 2011 to 2017

Profile		Food (20)	Humans (23)	N N=43
I	SUL/COL/MER/[CFT]/AMP/[CIP]/TET	0	1	1
II	SUL/COL/MER/AMP/CIP/TET	0	1	1
III	SUL/COL/MER/[CFT]/AMP/TET	1	1	2
IV	SUL/COL/MER/CFT/AMP/[CIP]	0	1	1
V	SUL/COL/[MER]/[CFT]/AMP/[CIP]	0	1	1
VI	SUL/COL/MER/CFT/AMP/TET	1	0	1
VII	SUL/COL/CFT/AMP/[CIP]/TET	0	2	2
VIII	SUL/[COL]/MER/AMP/[CIP]/TET	0	1	1
IX	SUL/COL/MER/CFT/AMP/[TET]	1	0	1
X	SUL/COL/MER/[CEF]/[AMP]/TET	0	1	1
XI	SUL/COL/MER/AMP/[CIP]	0	3	3
XII	SUL/COL/MER/AMP/TET	0	1	1
XIII	SUL/MER/CFT/AMP/[CIP]	1	0	1
XIV	SUL/COL/AMP/[CIP]/TET	2	0	2
XV	SUL/COL/MER/[CFT]/[AMP]	1	3	4
XVI	SUL/COL/[CFT]/AMP/TET	1	0	1
XVII	SUL/[COL]/MER/AMP/TET	1	0	1
XVIII	SUL/COL/MER/CFT/TET	0	2	2
XIX	SUL/COL/MER/CFT/[AMP]	0	1	1
XX	SUL/COL/MER/[CFT]/AMP	1	0	1
XXI	SUL/COL/MER/[CFT]	3	2	5
XXII	SUL/COL/MER/[CIP]	1	0	1
XXIII	SUL/MER/[CFT]/[CIP]	2	0	2
XXIV	SUL/COL/[MER]/[CFT]	1	0	1
XXV	SUL/[COL]/MER/AMP	1	0	1
XXVI	SUL/COL/CFT/TET	0	1	1
XXVII	SUL/MER/[CIP]	1	1	2
XXVIII	SUL/COL/MER	1	0	1

Different shade in gray in the table indicates the group of profiles with statistical difference in relation to the white ($p<0.05$ – Fischer test).

Suplementary Table S2: Identification and isolation data of ST strains from food and infected individuals in Brazil, from 2011 to 2017.

Strains	Isolation				
	**Year**	**Source**	**Local**	**Sample**	**Resistance Profile**
1T	2015	Human	RS	Feces	XI
2T	2015	Human	RS	Feces	II
3T	2012	Food	BA	Raw salada	XXI
4T	2012	Food	RS	Chilled beef	XXI
5T	2017	Human	RS	Blood	XXI
6T	2013	Food	SP	Pork meat	III
7T	2014	Human	SC	Feces	IV
8T	2014	Human	SC	Feces	XII
9T	2012	Food	BA	Raw salada	XXII
10T	2014	Human	RS	Feces	XI
11T	2014	Food	RS	PSM[2]	XXVIII
12T	2016	Food	RS	Turkey	XXVII
13T	2015	Human	RS	Feces	XI
14T	2012	Human	RS	Feces	V
15T	2012	Human	RS	Blood	I
16T	2011	Food	GO	Poultry	XXIII
17T	2017	Food	MG	Cheast fillet	XIII
18T	2017	Food	MG	MSM[3]	XIV
19T	2017	Food	MG	Swine ear	XIV
20T	2015	Human	RS	Feces	XXVII
21T	2016	Food	SC	MSM[2]	XXIII
22T	2011	Food	PR	Poultry	XV
23T	2014	Food	MG	Pork sausage	XVI
24T	2016	Human	RS	Feces	III
25T	2014	Human	RS	Feces	XV
26T	2015	Food	MG	Poultry	XXI
27T	2011	Food	RS	Poultry	VI
28T	2014	Food	MG	RF[3]	XVII
29T	2011	Human	RS	Feces	VII
30T	2015	Human	RS	Feces	XVIII
31T	2012	Food	MT	FPM[4]	XXIV
32T	2013	Food	SC	Chicken carcass	XXV
33T	2016	Human	GO	Abscess Sec.[6]	XXI
34T	2016	Human	RS	Feces	XVIII
35T	2014	Human	RS	Feces	XV
36T	2015	Human	RS	Feces	XIX
37T	2017	Human	RS	Feces	XV
38T	2012	Human	RS	Feces	VIII
39T	2013	Food	SC	Poultry	IX
40T	2011	Food	MT	Frozen fish	XX
41T	2013	Human	MG	Feces	XXVI
42T	2013	Human	RS	Blood	X
43T	2011	Human	RS	Feces	VII

[1]PSM = potato salad with mayonnaise; [2]MSM = mechanically separated meat; [3]RF = residue flour; [4]FPM = frozen pork meat; [6]Sec. = secretion.
